# Commentary: Comparative efficacy and safety of non-pharmacological interventions on treatment-induced xerostomia in patients with head and neck cancer: a systematic review and network meta-analysis

**DOI:** 10.3389/fonc.2026.1744455

**Published:** 2026-03-06

**Authors:** Sargon Shazo

**Affiliations:** Department of Oral and Maxillofacial Surgery, College of Dentistry, University of Duhok, Duhok, Iraq

**Keywords:** meta-analysis, photobiomodulation, radiotherapy, sialagogues, xerostomia

## 1 Introduction

I read with interest the recently published network meta-analysis (NMA) evaluating interventions for treating radiation-induced xerostomia in patients with head and neck cancer (1). The authors are to be congratulated for embarking on a methodologically intensive synthesis of comparative interventions aimed at this clinically burdensome sequela of radiotherapy. Because xerostomia considerably impairs patients’ quality of life and adherence to oncologic treatment regimens, systematic efforts toward identifying optimal interventions are of utmost relevance. On scrutiny, however, several important issues arise with respect to heterogeneity among the included interventions.

## 2 Commentary and discussion

While some of these issues were acknowledged as limitations in the original article, their methodological and clinical implications warrant further elaboration, particularly given the reliance on surface under the cumulative ranking curve (SUCRA)-based hierarchies for informing practice.

### 2.1 The need for further distinguishing between preventive and therapeutic intentions

With explicit inclusion criteria of patients developing xerostomia post-radiation in the study (1), trials investigating both preventive (2–4) and therapeutic modalities were included in the NMA without specification of their mechanistic or clinical intent. For example, photobiomodulation, pilocarpine, and acupuncture could have been administered as prophylaxis before or during radiotherapy or as treatment for established xerostomia. This conflation undermines the internal validity of the pooled estimates, as these strategies target divergent pathophysiological windows: prophylaxis targets the prevention of insult to the salivary glands, while treatment involves the modulation of residual gland function or neurostimulation following injury.

### 2.2 The difficulty of differentiating mixed xerostomia etiologies

Another methodological concern in this meta-analysis is the inclusion of studies evaluating xerostomia of other etiologies rather than radiation, introducing significant clinical and pathophysiological heterogeneity. For instance, Aagaard et al. (5) enrolled predominantly patients with Sjögren’s syndrome or drug-induced xerostomia, with only ~9% (4/43) having received head-and-neck radiotherapy. Similarly, Fidelix et al. (6) studied xerostomia in primary Sjögren’s syndrome, while Sugaya et al. (7) and Barbosa et al. (8) investigated interventions in cohorts with burning mouth syndrome (BMS). These etiologies differ substantially: both Sjögren’s syndrome and post-radiation xerostomia involve progressive glandular destruction—autoimmune-mediated lymphocytic infiltration in the former, acinar cell ablation and fibrosis in the latter—whereas drug-induced dry mouth reflects transient pharmacologic inhibition. BMS, conversely, is largely neuropathic, often presenting with normal stimulated salivary flow and representing a disorder of sensory perception rather than secretory failure.

Pooling these mechanistically disparate conditions undermines the NMA’s transitivity assumption and compromises the interpretability of SUCRA-derived rankings. Treatment efficacy is likely condition-specific; for example, low-level laser therapy showed no benefit in Sjögren’s syndrome but modest gains in idiopathic BMS. Without stratification by etiology, the ranking hierarchy carries the risk of misguiding clinicians.

### 2.3 Clinical applicability of composite outcomes

Pooling therapeutic and preventive intervention outcomes across the studies, along with different etiological backgrounds of the studied conditions, presents violations of transitivity in the assumptions of NMA. The absence of subgroup stratification based on the timing of therapy obscures the clinical decision pathway: a radiation oncologist who wants to prescribe a prophylactic agent cannot meaningfully extrapolate from the outcomes of post-radiation management, and *vice versa*. In clinical oncology, the temporal sequencing of interventions is inseparable from the interpretation of efficacy.

### 2.4 Methodological implications and certainty of evidence

The incorporation of heterogeneous study purposes may weaken confidence in the SUCRA results, a pillar of NMA-derived hierarchies. Furthermore, indirectness, according to the GRADE framework, is greatly increased when trials from different populations and clinical settings are assessed together.

### 2.5 Clinical perspective and practical experience

In our head and neck oncology practice, xerostomia remains a major determinant of long-term quality of life after radiotherapy. Management is multimodal and individualized; we incorporate preventive counseling during treatment planning, early recognition of salivary hypofunction through subjective and objective assessments, and stepwise therapeutic escalation. Preliminary measures include improvement of oral hygiene, saliva substitutes, and making dietary modifications. In patients with gland hypofunction, strategies such as chewing gum, gustatory stimulation, and sialagogues, when clinically appropriate, are considered, while low-level laser therapy is selectively considered in patients with residual gland function and applied at time points where therapeutic benefit is biologically plausible. Importantly, the timing of intervention—whether preventive or therapeutic—influences both expected efficacy and outcome interpretation, emphasizing the need for evidence syntheses to clearly distinguish therapeutic intent to ensure meaningful clinical applicability of comparative rankings.

### 2.6 Recommendations for future NMAs

We recommend the clear *a priori* specification of inclusion criteria according to the therapeutic phase—preventive versus therapeutic—for NMAs on radiation-induced toxicities. Analyses should be performed in separate networks or, at least, hierarchical subgroup analyses, to maintain methodological homogeneity. The definition of the xerostomia endpoints (e.g., patient-reported outcomes vs. sialometry) needs harmonization to ensure construct validity.

## 3 Conclusion

In summary, this commentary underscores the importance of methodological clarity when evaluating interventions for radiation-induced xerostomia with a focus on xerostomia of a post-radiation etiology, furthermore highlighting that a “one-size-fits-all” analysis may obscure contextual nuances. This NMA represents an important step toward consolidating the evidence for the management of radiation-induced xerostomia. However, its clinical applicability is limited by the fact that studies across the prevention–treatment continuum and different backgrounds are undifferentiated. Future efforts are encouraged to adopt stratified or phase-specific approaches to better inform clinical decision-making grounded in pathophysiological context and therapeutic intent.
